# Intelligent Physical Robots in Health Care: Systematic Literature Review

**DOI:** 10.2196/39786

**Published:** 2023-01-18

**Authors:** Rong Huang, Hongxiu Li, Reima Suomi, Chenglong Li, Teijo Peltoniemi

**Affiliations:** 1 Department of Management and Entrepreneurship Turku School of Economics University of Turku Turku Finland; 2 Department of Information and Knowledge Management Tampere University Tampere Finland

**Keywords:** intelligent physical robot, artificial intelligence, health care, literature review

## Abstract

**Background:**

Intelligent physical robots based on artificial intelligence have been argued to bring about dramatic changes in health care services. Previous research has examined the use of intelligent physical robots in the health care context from different perspectives; however, an overview of the antecedents and consequences of intelligent physical robot use in health care is lacking in the literature.

**Objective:**

In this paper, we aimed to provide an overview of the antecedents and consequences of intelligent physical robot use in health care and to propose potential agendas for future research through a systematic literature review.

**Methods:**

We conducted a systematic literature review on intelligent physical robots in the health care field following the guidelines of PRISMA (Preferred Reporting Items for Systematic Reviews and Meta-Analyses). Literature searches were conducted in 5 databases (PubMed, Scopus, PsycINFO, Embase, and CINAHL) in May 2021, focusing on studies using intelligent physical robots for health care purposes. Subsequently, the quality of the included studies was assessed using the Mixed Methods Appraisal Tool. We performed an exploratory content analysis and synthesized the findings extracted from the included articles.

**Results:**

A total of 94 research articles were included in the review. Intelligent physical robots, including mechanoid, humanoid, android, and animalistic robots, have been used in hospitals, nursing homes, mental health care centers, laboratories, and patients’ homes by both end customers and health care professionals. The antecedents for intelligent physical robot use are categorized into individual-, organization-, and robot-related factors. Intelligent physical robot use in the health care context leads to both non–health-related consequences (emotional outcomes, attitude and evaluation outcomes, and behavioral outcomes) and consequences for (physical, mental, and social) health promotion for individual users. Accordingly, an integrative framework was proposed to obtain an overview of the antecedents and consequences of intelligent physical robot use in the health care context.

**Conclusions:**

This study contributes to the literature by summarizing current knowledge in the field of intelligent physical robot use in health care, by identifying the antecedents and the consequences of intelligent physical robot use, and by proposing potential future research agendas in the specific area based on the research findings in the literature and the identified knowledge gaps.

## Introduction

### Background

With the development of artificial intelligence (AI), physical robots with intelligent capabilities based on AI (hereinafter intelligent physical robots) have been applied in the health care context to expand the digitization of health care work processes and increase the use, fairness, and cost-effectiveness of health care services, such as in smart health care services [1,2], including telemedicine [3], ambient-assisted living [4], intelligent health management [5], psychotherapy [6], and companionship [7].

The use of intelligent physical robots has attracted the attention of scholars, and various studies have examined the use of intelligent physical robots in health care from different angles. For instance, some studies have investigated the use of intelligent physical robots from the perspective of anthropomorphic design and features [8], social interaction [9], personality [10], and intelligence function [11] in various health care contexts, such as nursing homes [12], hospitals [13], psychiatric clinics [14], and patients’ homes [15,16]. Another research stream has mainly investigated how robot use affects individuals, such as users’ mood and behavior [17], user attitudes toward robots [18], and health promotion [19,20]. Although prior studies provide an understanding of intelligent physical robot use in health care, each study only examines the topic from a specific point of view. A couple of studies have attempted to provide an overview of robots in the health care context, but these studies have focused on either specific health care contexts or specific robot devices [21]. For instance, Sarker et al [21] reviewed the literature to identify how intelligent robots can help health care professionals fight the COVID-19 pandemic, whereas Vélez-Guerrero et al [22] focused on AI-based wearable robotic exoskeletons for rehabilitation by reviewing relevant articles. These studies failed to provide an overview of the use of intelligent physical robots in the general health care context.

In addition, prior literature has stated that it is important to understand the antecedents and consequences of innovative IT use to improve both user acceptance and IT performance [23,24]. Thus, it is imperative to obtain an overview of the antecedents and consequences of intelligent physical robot use in health care based on a literature review, which will provide state-of-the-art knowledge for both scholars and practitioners.

To fill this research gap, we aimed to provide an overview of the research on the use of intelligent physical robots in health care through a systematic literature review, especially to identify its antecedents and consequences. In addition, we aimed to identify the potential future research agendas in the field to guide scholars’ future research in the field.

### Related Work

A widely used definition of a robot is provided by Nejat et al [25]: autonomous or semiautonomous artificial objects and devices programmed to act and perform tasks in their environment. Depending on the nature of the embodiment, robots can be divided into physical robots with visually observable bodies (eg, the humanoid robot *Pepper* and the animal-like robot *Paro*) and internet-based robots generated through computer algorithms that respond to users in natural language (eg, chatbots and animation robots). Many studies have shown that physical robots are more expressive than internet-based robots in terms of interactive functions and physical embodiment [26,27]. From a functional point of view, the intelligent capabilities of physical robots are mainly reflected in three aspects: (1) perceiving surrounding informational and environmental changes, (2) thinking and learning, and (3) handling various complex tasks autonomously and proactively [28].

Some research has focused on the use of intelligent physical robots at the individual level, such as among patients and health care professionals. Fasola and Matarić [29] investigated the intrinsic motivations for older adults to engage in physical exercise using socially assistive physical robots and found that users’ perceptions of the enjoyment, usefulness, helpfulness, social attraction, and social presence in robot use motivated their use. Kuo et al [30] identified gender differences in patients’ attitudes and reactions toward intelligent physical robots in home care centers. Chang et al [31] examined how intelligent physical robots can reduce nurses’ workloads and turnover intentions in hospitals. Mettler et al [32] found that different professionals’ acceptance of and resistance to physical service robots are determined by their shared beliefs and concerns as well as their perceived affordance of physical service robots. Some studies have also found that robot design is closely linked to individuals’ use of robots in health care, such as anthropomorphism, social capabilities, and intellectual capabilities [33,34]. Meanwhile, some studies have investigated the use of intelligent physical robots from an organizational point of view. For example, Lee et al [35] found that management support facilitated the adoption of intelligent physical robots in an organization. Intelligent physical robots have also been found to be an effective means for hospitals to improve cost-effectiveness and health care service delivery [36,37].

Although prior studies offer important insights into intelligent physical robot use in health care, these studies have mainly provided knowledge of its antecedents and consequences from different perspectives and cannot yet provide an overview of its use in health care, which gives rise to the need for this study.

## Methods

### Overview

We conducted a systematic literature review following the PRISMA (Preferred Reporting Items for Systematic Reviews and Meta-Analyses) guidelines. The details of the PRISMA guidelines are presented in Multimedia Appendix 1 [38]. The literature review comprised the following steps: (1) database search; (2) eligibility criteria; (3) study selection and screening; (4) quality appraisal of studies; and (5) data extraction, analysis, and synthesis.

### Database Search

We conducted database searches in the PubMed, Scopus, PsycINFO, Embase, and CINAHL electronic databases in May 2021. These 5 electronic databases in health, nursing, biomedicine, and psychology were selected to search for eligible studies as widely as possible. The following search terms were used to search titles and abstracts of articles and to find subject-specific articles for the literature review: “healthcare,” “health care,” “nursing, robot*,” and “bot.” The search string had wide coverage to avoid missing any research of interest. In our search, there were no restrictions on publication time or study design. Studies published in languages other than English were excluded. The search strategy for each database is shown in Textbox 1, and the detailed explanation of these search strategies is provided in Multimedia Appendix 2.

**Databases and search terms.**

- PubMed: ([(healthcare[Title/Abstract]) OR (“health care”[Title/Abstract])] OR [nursing(Title/Abstract)]) AND ([robot*(Title/Abstract)] OR [bot(Title/Abstract)])
- Scopus: (TITLE-ABS [healthcare] OR TITLE-ABS [“health care”] OR TITLE-ABS [nursing]) AND (TITLE-ABS [robot*] OR TITLE-ABS [*bot])
- PsycINFO: (abstract [health care] OR abstract [“health care”] OR abstract [nursing]) AND (abstract [robot*] OR abstract [*bot])
- Embase: robot*:ab,ti OR (bot:ab,ti AND healthcare:ab,ti OR “health care”:ab,ti OR nursing:ab,ti
- CINAHL: (AB [healthcare] OR AB [“health care”] OR AB [nursing]) AND (ABS [robot*] OR AB [*bot])

### Eligibility Criteria

Studies were selected for this literature review according to the following inclusion criteria: (1) studies examining at least 1 physical robot with intelligent capabilities to assist users in completing tasks; (2) studies examining robots for health care purposes, which means they aim to use robots to promote or monitor health, to assist in tasks that are difficult to perform because of health problems, or to prevent further health decline [39]; (3) peer-reviewed, full-length articles published in journals, conferences, and books; and (4) studies published in English.

Some studies were excluded from this literature review because of the following reasons: (1) studies were not published in English; (2) studies were incomplete or non–peer-reviewed; (3) studies examining robots without embodied physical appearance (eg, conversational agents, robotic process automation, and robotic software); (4) studies examining robots without intelligent capabilities, including robots mainly for automation that allow users to choose from predefined options or robots that cannot adapt to dynamic and uncertain environments; (5) studies examining the design and development of robots without the actual use implementation and evaluation of robots; and (6) studies in which robots were not implemented for the purpose of health care (such as using robots to promote or monitor health, to assist in tasks that are difficult to perform owing to health problems or to prevent further health decline).

### Study Selection and Screening

All relevant studies identified by database searches were downloaded and stored in the reference management software EndNote (version X9; Clarivate), which automatically eliminated duplicates. Initial selection of the studies was performed independently by the first and second authors by screening the titles and abstracts of the identified articles. All disagreements were resolved through discussion. In a second screening step, the full texts of the relevant articles were independently examined by the first and second authors according to the inclusion and exclusion criteria. All disagreements were resolved with joint discussion and final agreement between the 2 authors.

### Quality Appraisal of Studies

To evaluate the methodological quality of the studies, the first and second authors appraised the quality of the selected articles independently according to the Mixed Methods Appraisal Tool (MMAT; version 2018) [40], which can be applied to evaluate empirical studies using 5 different research methods: qualitative methods, quantitative randomized controlled trials, quantitative nonrandomized trials, quantitative descriptive methods, and mixed methods. Each category is assessed by 5 different quality parameters, with final scores of 1 to 2=low quality, 3=moderate quality, or 4 to 5=high quality. All disagreements were resolved through discussions between the first and second authors.

### Data Extraction, Analysis, and Synthesis

The following information was extracted for each eligible article by the first and second authors: publication type, titles, authors, publication year, research method, theoretical base, robotic platform, context, and main findings. Any disagreement was resolved through a discussion with the entire research team.

We aimed to provide an overview of the antecedents and consequences of intelligent physical robot use in health care. As there was no existing theory to serve as a framework for our study, we performed an exploratory conventional content analysis [41] to analyze the included articles. We captured a list of terms or phrases regarding the antecedents or the consequences of intelligent physical robot use based on our reading of the included articles and coded them. We first conducted the coding based on our reading of 15 articles and set up the preliminary codes. Next, we coded the remaining articles using these codes. We added new codes when we found new antecedents or consequences that were not in the existing codes.

The codes of the antecedents and the consequences of intelligent physical robot use were sorted into categories based on their characteristics, and a hierarchical structure of the antecedents and the consequences was established to guide the synthesis of the findings from the included articles. Specifically, the antecedents were categorized into individual-, organization-, and robot-related factors, and the consequences consisted of both non–health-related consequences and consequences for health promotion. The non–health-related consequences include emotional outcomes, attitude and evaluation outcomes, and behavioral outcomes of technology use, whereas the consequences for health promotion include physical health promotion, mental health promotion, and social health promotion. Finally, we discussed and finalized the results of the data synthesis.

## Results

### Overview

This section includes a description of the articles included in this study, the quality assessment of these included articles, the publication year, and the publication sources of these included studies. We have summarized the applied research methods and theories, research contexts, robotic devices, and target users in these studies. Finally, we synthesized the antecedents and consequences of intelligent physical robot use in health care based on the included studies and proposed an integrative framework for the antecedents and consequences of physical robot use in health care to provide an overview of physical intelligent robot use in health care based on the findings of the included studies.

### Articles Included in the Review

**Figure 1:**
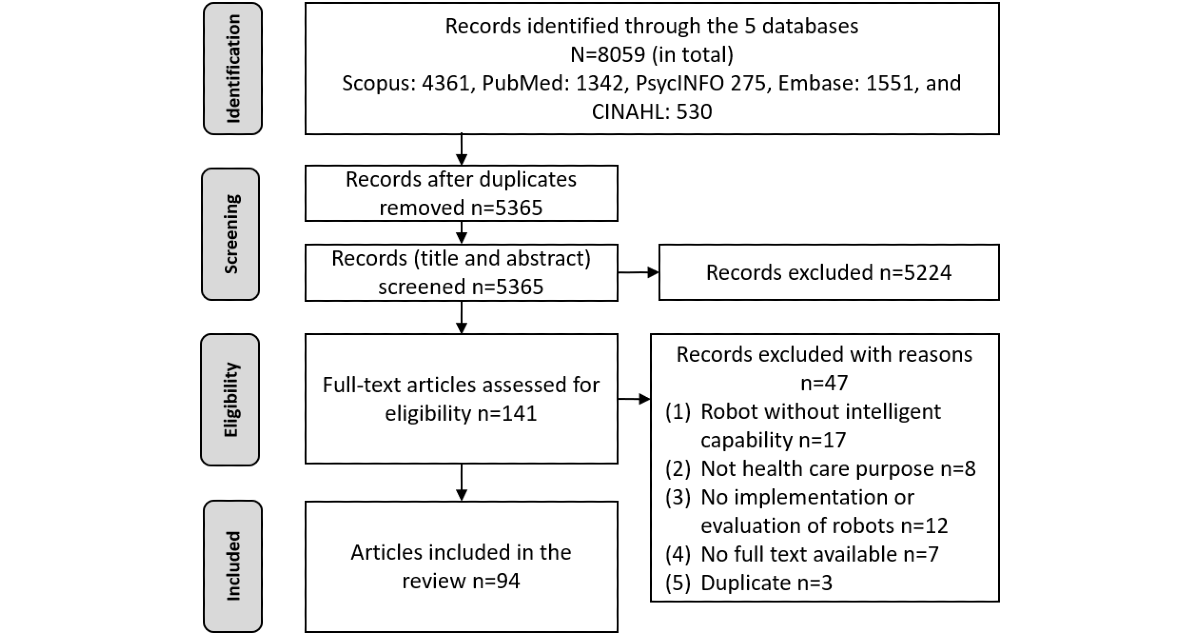
PRISMA (Preferred Reporting Items for Systematic Reviews and Meta-Analyses) flowchart.

Figure 1 shows the PRISMA flowchart for the study selection process. Initially, the database search identified 8059 articles. Some articles were duplicated in different databases. After removing the duplicate articles retrieved from different databases, 5365 entries were left. Of the remaining 5365 articles, 5224 (97.37%) articles were excluded from the literature review after title and abstract screening as they did not meet the eligibility criteria to be included in this study. After full-text screening of the 141 remaining articles, 47 more were excluded. Thus, 94 articles were included in this systematic review. The details of the 94 selected articles are provided in Multimedia Appendix 3 [3-6,10-18,20,30-37,42-113].

We performed a methodological quality assessment of the 94 included studies using the MMAT. Among these 94 studies, 62 (66%) were rated as high quality, 24 (26%) as moderate quality, and 8 (8%) as low quality. Although these 8 studies have methodological limitations, they provided some new insights in some specific research contexts regarding intelligent physical robot use, such as in cognitive interventions and physical rehabilitation, which have been rarely covered by other studies. Thus, we included the 8 articles with low methodological quality in this systematic literature review. The details of the quality assessment are provided in Multimedia Appendix 4 [3-6,10-18,20,30-37,42-113].

The 94 articles included in the literature review were published between 2009 and May 2021 (Table 1). More than half of these articles were published after 2017. Among them, 66% (64/94) were published in journals and the rest in proceedings of international conferences.

**Table 1:** Distribution of articles by publishing year (to May 2021; n=94).

Publication year	Publications, n (%)
2009	2 (2)
2010	3 (3)
2011	3 (3)
2012	2 (2)
2013	9 (10)
2014	10 (11)
2015	6 (6)
2016	3 (3)
2017	11 (12)
2018	10 (11)
2019	10 (11)
2020	18 (19)
2021	7 (7)

### Research Methods, Theories, and Contexts Applied in the Included Studies

With regard to the methods used in the included studies, the experiment was the dominant research method; 55% (52/94) of studies opted for an experimental approach. Surveys (16/94, 17%), interviews (10/94, 11%), and mixed methods (10/94, 11%) were also used to gauge the representativeness of individual views and experiences, accounting for 38% (36/94) of the included studies. Among the studies that applied mixed methods, 30% (3/10) used focus groups and questionnaires, 60% (6/10) used interviews and questionnaires, and 10% (1/10) used Q-methodology, which combines qualitative and quantitative data analysis methods. Some studies applied case studies (3/94, 3%), observational studies (2/94, 2%), and ethnographic methods (1/94, 1%).

Different theories have been applied in these included studies to investigate robot use in the health care context. Two popular technology use models, the technology acceptance model and the unified theory of acceptance and use of technology, have been widely applied in some studies to investigate the use of robots in the health care context [15,42-45]. In addition, other theories regarding user behavior, such as applied behavior analysis [46], the theory of planned behavior [47,48], IT affordance [32], and activity engagement theory [49], have been used to explain user behavior regarding robot use. In mental health contexts, some psychology-related theories have been applied to understand robot use from a psychological viewpoint, such as the theory of mind [44,45], emotional well-being [50], emotional appraisal [51], and the capability approach [52]. Moreover, some studies have applied nursing-related theories to investigate the use of robots from a health care professional perspective, such as the transactive relationship theory of nursing [53], professional task engagement [31], job satisfaction [31], and person-centered care [14].

Robots have been widely studied in care facilities, including older care facilities [12,54,55], long-term care facilities [13,50], and mental care facilities [56,57]. The use of robots in professional medical contexts has also been studied, such as in hospitals [31], outpatient clinics [58], and simulation laboratories [49]. Health care robots serving residents and their caregivers in retirement villages [20] and communities [59] are popular contexts in the included studies. Several studies have investigated the use of health care robots in participants’ homes [30,60] and universities [61]. Multimedia Appendix 5 [3-6,10-18,20,30-37,42-95,97-113] describes the contexts of the included studies.

### Robotic Devices and Target Users

As shown in Multimedia Appendix 6 [3,5,6,10,12, 14,16,17,20,30,33,34,36,37,42,45,46,49-54,56-59,61-64,66-71,73, 74,77,78,83,85-88,90-101,103,105-107,109,111], a total of 33 different robotic platforms were investigated in the included studies. Among them, the most popular robotic platforms are Pepper, Paro, Nao, iRobiQ, and Healthbot. These robots are usually equipped with a variety of sensors to support their performance of different functions, such as sight, sound, balance, and touch [65,66,114,115]. In terms of their physical characteristics, these robotic devices can be divided into mechanoid, humanoid, android, and animalistic.

Twelve of the robotic devices mentioned in 18% (17/94) of the included studies were mechanoid robots, which were described as having a mechanical appearance and no overtly human-like features, such as a kiosk with a screen [116]. For example, Lio looks like a robotic arm placed on top of a mobile platform and can provide humans with daily life assistance such as navigation, grasping, and monitoring [88].

In addition, 21% (20/94) of studies investigated the use of animalistic robots. These animal-like robots have a cute appearance and are mainly used for psychological and emotional interventions for older patients with mental illnesses. For example, a robotic baby seal named Paro has been widely used to accompany and help older people with cognitive and psychological impairments [56,57].

Among the included studies, 28% (26/94) of studies investigated humanoid robots, which usually have physical structures and movement patterns similar to those of humans [62,63]. Furthermore, 4% (4/94) of studies examined the use of android robots, which have a more realistic human-like appearance than humanoid robots [10,16,51,64].

The target users studied in the included articles can be classified into 2 types: end customers and professionals. Specifically, regarding end customers, 40% (38/94) of studies investigated robot use among older adults, such as the impact of robots on their physical health [12], cognitive health [55], and quality of life [3]. In addition, 21% (20/94) of studies emphasized patients as end customers, such as hospitalized patients (4/94, 4%) [10,13,34,36], people with cognitive impairment (10/94, 11%) [54], children with chronic diseases (4/94, 4%) [46,59,65,105], and people with limited arm or leg mobility (2/94, 2%) [66,100]. Furthermore, 14% (13/94) of studies investigated the general public’s awareness of health care robots, including healthy adults (5/94, 5%) [5,12,16,30,49,80] and university students and employees (8/94, 9%) [10,48,51,61,64,68,71,104]. Overall, 4% (4/94) of studies focused on patients’ relatives [75,97,105,111]. Health care professionals include nurses (20/94, 21%) [31], personal care and home care workers (16/94, 17%) [43], medical doctors (5/94, 5%) [6,15,18,32,44], management personnel (11/94, 12%) [32], and other staff (8/94, 9%) [11,15,18,44,67,70,72,76]. Multimedia Appendix 7 [3-6,10-18,30-37,42-59,61-87,89-113] presents details of the target users in the included studies.

### Antecedents of the Use of Robots in Health Care

#### Overview

Of the 94 included studies, 37 (39%) investigated the factors of end customers and health care professionals from an individual perspective, which were listed as individual-related antecedents. In total, 13% (12/94) of studies investigated the internal characteristics or resources of health care organizations from an organizational perspective, and these were listed as organization-related antecedents; 21% (20/94) of studies investigated the design and technical characteristics of robotics applications from a technology developer perspective, which were listed as robot-related antecedents (Multimedia Appendix 8 [3-6,10,11,14,16,17,20,30,32-36,42-45,48,51-53,56,61,63,64, 66-68,71-76,80-89,92,102-104,112]).

#### Individual-Related Factors

In the health care context, the use of robots is influenced by demographic, psychological, and experience-related factors of individual users, including both end customers and health care professionals [68-76].

Overall, 10% (9/94) of studies investigated the influence of demographic factors, including age, gender, nationality, and education, on robot use in health care [30,43,44,68,71,72,75,76,85]. Specifically, 4% (4/94) of studies reported that male and highly educated end customers are more likely to accept intelligent physical robots in health care [30,68,71,85], whereas 3% (3/94) of studies found that female health care personnel tend to have more concerns about the technology and safety issues surrounding robot use [44,72,76]. The findings of 3% (3/94) of other studies have shown that young end customers have a more positive attitude toward health care robots [30,44,68]. Furthermore, nationality was found to have a significant impact on end customers’ and health care professionals’ opinions and preferences regarding the design, function, and roles of intelligent physical robots in health care in 2% (2/94) of studies [43,75].

In total, 31% (29/94) of studies examined the impact of psychological factors on robot use. Fifteen studies reported that end customers’ psychological states (eg, preinteraction emotions, motivations, and attitudes) could affect their willingness to use intelligent physical robots in health care [17,45,73,74]. End customers’ perceptions, such as perceived intelligence, anthropomorphism, safety, usefulness, and ease of use, were found to lead to human-robot interaction in 6% (6/94) of studies [5,6,20,45,68,77]. The findings of 13% (12/94) of studies have shown that health care professionals’ emotions, attitudes, and ethical perceptions are associated with their use of robots in the workplace [44,53,78-81].

Overall, 9% (8/94) of studies examined the impact of experience-related factors on the use of robots [20,35,44,45,72,76,82,83]. Specifically, 3% (3/94) of studies found that end customers’ experiences of using intelligent physical robots or of exposure to intelligent physical robots influence their acceptance of intelligent physical robots in health contexts [20,45,82], whereas the findings of other 5% (5/94) of studies showed that health care professionals’ working experience with innovative technologies or robotics and their managerial experience are positively associated with their acceptance of intelligent physical robots at work [35,44,72,76,83].

#### Organization-Related Factors

In addition to individual factors, 13% (12/94) of studies have analyzed the environment- and resource-related antecedents of robot use at the organizational level to examine physical intelligent robot use in various health care organizations.

In total, 5% (5/94) of studies examined the impact of environment-related factors on physical intelligent robot use among end customers and health care professionals [14,32,33,84,102]. For instance, 3% (3/94) of studies found that building structure, layout, and decoration with regard to robot use are associated with health care professionals’ acceptance of intelligent physical robots in hospitals and older care facilities [32,33,84]. Overall, 2% (2/94) of studies reported that the atmosphere (such as lower noise levels, comfortable room temperature, and balanced lighting) in which intelligent physical robots are used in mental care facilities and hospitals can affect both nurses’ and patients’ emotions in robot use as well as their use experience [14,84].

Moreover, 14% (13/94) of studies highlighted the role of resource-related factors in explaining intelligent physical robot use among health care professionals in health care organizations. Specifically, 4% (4/94) of studies found that top management support could create a positive environment for intelligent physical robot use among health care professionals [44,72,83,112]. In total, 6% (6/94) of studies reported that personnel readiness factors, such as training and upskilling of personnel, could affect intelligent physical robot use among health care professionals [14,35,44,67,83,104]. Overall, 3% (3/94) of studies discussed that IT infrastructure (eg, Bluetooth, helpdesk availability, and Wi-Fi connection) could be an important factor affecting health care professionals’ willingness to accept and use robots in work, thus facilitating the optimal operation of health care services [32,83,84].

#### Robot-Related Factors

Among the included studies, 21% (20/94) of studies have examined physical intelligent robot use in various health care organizations from the perspective of robots, mainly from the characteristics of the design and functions of intelligent physical robots, such as anthropomorphism, appearance, intelligence, and voice.

In total, 18% (17/94) of studies examined the effects of robot design on intelligent physical robot use among end customers and health care professionals. Specifically, 7% (7/94) of studies discussed how the appearance (such as anthropomorphism, body size, gender, and ethnicity) of intelligent physical robots affects the acceptance of intelligent physical robots in various health care services among end customers [16,34,48,61,85,86,112]. Overall, 10% (9/94) of studies reported that a robot’s empathetic voice, humor, or extroverted personality could positively influence its use among end customers and personal care workers [6,10,48,61,63,64,67,87,88].

In total, 10% (9/94) of studies investigated how the functions of intelligent physical robots affect their use among end customers and health care professionals. Specifically, 6% (6/94) of studies found that the cognitive, navigation, interactive capabilities, and automatic response of intelligent physical robots could lead to end customers’ and health care professionals’ satisfaction with the robots [10,33,74,83,87,88]. Overall, 3% (3/94) of studies discussed the negative impact of software failures (such as technical language issues, operating noise, and sensor failure) on health care professionals’ experience of intelligent physical robot use [56,84,89].

### Consequences of Robot Use in Health Care

#### Overview

Intelligent physical robots in health care could lead to both non–health-related (emotional outcomes, attitude and evaluation outcomes, and behavioral outcomes) consequences and consequences for (physical, mental, and social) health promotion. Of the 94 included studies, 72 (77%) examined users’ emotional outcomes, attitude and evaluation outcomes, and behavioral outcomes in intelligent physical robot use (non–health-related consequences) and 39 (41%) studies investigated how intelligent physical robots can promote end users’ physical, mental, and social health (consequences for health promotion; Multimedia Appendix 9 [3-6,10-17, 20,30-37,43,46,48-50,52-63,65-76,82,83,85-105,107-113]).

#### Non–Health-Related Consequences

The emotional outcomes investigated in 9% (8/94) of studies have shown that end customers and health care professionals could have positive emotions when interacting with intelligent physical robots, such as pleasure [90], satisfaction [13,31], likeability [5], and enjoyment [71,91]. Meanwhile, 2% (2/94) of studies mentioned patients’ wariness when encountering the humanoid features of intelligent physical robots [34,61].

Furthermore, 46% (43/94) of studies examined end customers’ and health care professionals’ attitudes and evaluation outcomes regarding intelligent physical robot use in health care, showing mixed findings [87,89]. Specifically, 27% (25/94) of studies reported positive attitudes [48,89] and acceptance [4,58] of intelligent physical robots in health care. Some negative opinions and concerns about robots’ appearance, limited capability, and social and ethical implications have also been reported among end customers and health care professionals in 17% (16/94) of studies [74,75,83,86]. Moreover, 18% (17/94) of studies have reported user preferences for particular robot functions (eg, fall detection, cleaning, and medication reminders) and design (eg, humanoid appearance and social capability) [12,35].

In total, 48% (45/94) of studies examined the behavioral outcomes of intelligent physical robot use, including willingness to use, willingness to interact, frequency and duration of use, task performance, and response and engagement with robots. Specifically, 14% (13/94) of studies investigated the willingness to use or interact with intelligent physical robots among end customers and health care professionals [89,95], 10% (9/94) of studies examined the frequency and duration of robot use among end customers [36,55,58,67,71,92,96,109,111], and 10% (9/94) of studies have explained how intelligent physical robot use could help end customers and health care professionals improve their performance regarding different health care tasks, such as health data collection, rehabilitation exercise, and cognitive games [12,13,46,62,66,82,91,101,109]. In addition, 21% (20/94) of studies have examined end customers and health care professionals’ responses and engagement with robots from a multifaceted view [83,98].

#### Consequences for Health Promotion

In total, 41% (39/94) of studies reported that intelligent physical robot use could promote end customers’ health and well-being from the perspectives of physical, mental, and social health. Furthermore, 13% (12/94) of studies reported physical health promotion with intelligent physical robot use among end customers, including improvement of physical health medication adherence, rehabilitation prompts, and independent life among patients [3,20,92]. For instance, 3% (3/94) of studies investigated how intelligent physical robot use could help with patients’ use of medication (eg, anodynes or aldazines) [3,20,99]. Another 3% (3/94) of studies examined how older adults can successfully engage in and complete rehabilitation exercises under the guidance of intelligent physical health care robots [12,53,92]. Moreover, 6% (6/94) of studies reported that intelligent physical robots could perform some daily living tasks for older patients (such as heavy lifting, logistics, and safety detection) and some basic nursing tasks (such as measuring vital signs and medication delivery), which could improve the ability and quality of independent living for people with chronic diseases or mobility impairments [6,68,82,93,105,109].

In total, 21% (20/94) of studies reported mental health promotion with intelligent physical robot use among end customers from different perspectives. Of these, 6% (6/94) of studies examined mental health promotion from the perspective of improving medication adherence [3,20,54,56,99,100] and 5% (5/94) of studies from the perspective of monitoring and improving mood changes in adult patients with mental health problems [10,17,57,59,107]. In addition, 5% (5/94) of studies investigated how intelligent physical robots can enhance the cognitive capabilities of children with autism [46,49,57,98,101]. Furthermore, 13% (12/94) of studies examined how intelligent physical robots could be applied in interventions to alleviate the behavioral and psychological symptoms of dementia, such as improving anxiety, agitation, and depression [20,100,102], as well as providing distractions and interrupting problematic behaviors [74]. However, 1% (1/94) of studies also indicated that the therapeutic effects of robotic intervention cannot be maintained over time [55].

Furthermore, 16% (15/94) of studies examined how intelligent physical robot use in health care can promote social health. For instance, 4% (4/94) of studies found that older patients improved their engagement in group activities guided by intelligent physical health care robots in an older care facility [6,50,92,109]. Health care robots have also been found to facilitate social connections between patients and their families, neighbors, and caregivers in the form of physical contact, eye contact, and verbal communication in the findings of 7% (7/94) of studies [55,56,74,94,103,106,107]. Overall, 6% (6/94) of studies reported that intelligent physical robots can serve as conversational partners in daily life, providing end customers with opportunities to communicate and confide, thereby alleviating the loneliness of some end customers who live alone [6,66,85,94,104,110].

### A Framework to Understand Intelligent Physical Robot Use in Health Care

**Figure 2:**
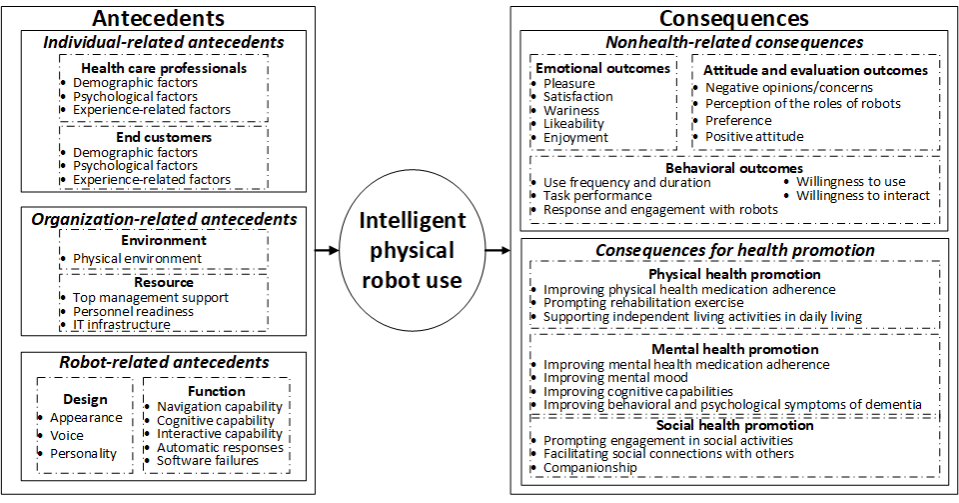
An integrative framework for understanding intelligent physical robot use in health care.

Various antecedents and consequences related to intelligent physical robot use in health care were identified based on reviewing the included articles. The factors related to individuals, organizations, and robots provide a multidimensional understanding of the factors determining intelligent physical robot use in health care, and the use of intelligent physical robots in health care can lead to both non–health-related (emotional, attitude and evaluation, and behavioral) outcomes and consequences for (physical, mental, and social) health promotion. A theoretical framework to present a holistic view of these factors is developed (Figure 2), which could guide future research in the field.

## Discussion

### Principal Findings

In this systematic review, we examined 94 studies that focused on intelligent physical robot use (eg, mechanoid, humanoid, android, and animalistic) in various health care contexts (eg, hospitals, personal care facilities, laboratories, and patients’ homes). The target users of intelligent physical robots in the health care context include end customers and health care professionals. We identified the antecedents of intelligent physical robot use at the individual level (eg, social and psychological factors), organizational level (environment and resources), and robot level (eg, robot design and functions). We also synthesized the consequences of intelligent physical robot use for individuals into non–health-related consequences (emotional outcomes, attitude and evaluation outcomes, and behavioral outcomes regarding technology use) and consequences (physical, mental, and social) for health promotion. We proposed an integrative framework of these antecedents and consequences to obtain a holistic overview of the intelligent physical robot use in the health care field.

In the health care context, social and psychological factors affect the use of intelligent physical robots at the individual level, including end customers and health care professionals [106,107]. These findings are consistent with prior research findings, which also found that social and psychological factors affect nurses’ and other health care and social care workers’ use of physical robots and internet-based robots [27,117]. Although only 2% (2/94) of studies examined cultural differences, these studies do provide evidence of cultural differences in user perception [43,75]. In addition to user characteristics, the design and functions of intelligent physical robots are also important factors affecting their use in the health care context, which is consistent with the findings on intelligent physical robot use in other contexts. For example, the anthropomorphic features and designs of intelligent physical robots have been argued to play important roles in explaining robot use among end customers in the context of hospitality and education [19]. Some organizational factors, such as the physical environment and relevant resources of health care organizations, have also been found to be important in intelligent physical robot use [44,83]. Among the included 94 studies, there are fewer studies examining the use of intelligent physical robots at the organizational level compared with the number of studies focusing on individual users [35,67].

Various non–health-related consequences of intelligent physical robot use in health care were identified in this study, including emotional, attitudinal, and behavioral outcomes. These findings provide a rich understanding of the non–health-related consequences of intelligent physical robot use in health care among different user groups with various robot use purposes across different contexts in health care [108,114,118]. The use of intelligent physical robots in health care could, to some degree, improve the physical, mental, and social health of individual users [3,74,90]. These findings are consistent with the results of a literature review focusing on intelligent physical robots in care delivery during the COVID-19 pandemic from an organizational perspective. This review also found that the use of intelligent physical robots could promote health during the COVID-19 pandemic, such as diagnosis, risk assessment, monitoring, telehealth care, disinfection, and service automation [21]. The findings of this study complement prior research by providing a view on the potential physical, mental, and social consequences of intelligent physical robot use among various groups across different health care scenarios, such as older care, emotional health care, cognitive improvement, social participation, quality of life, and well-being [88,110].

Some studies have mentioned the following challenges and barriers in intelligent physical robot use in health care: perceived fear, distrust, and uncomfortable feelings caused by robots’ anthropomorphic design, technical barriers, and limited intelligence capabilities [111]. As Vélez-Guerrero et al [22] proposed, there is a need to improve the weight, operation mode, and control systems of robotic exoskeletons from the view of robot design to address certain challenges and barriers related to robot design. Clearly, more research is needed to address different challenges and barriers in intelligent physical robot use in health care and to make intelligent physical robots serve society in a health care setting.

### Future Research Agenda

#### Overview

On the basis of our analysis of the included studies, we found that the literature on intelligent physical robots in the health care context is still in its nascent stage. There is great potential for the development of research on the conceptualization of intelligent physical robots and the breadth and depth of intelligent physical robot use in health care by applying different research methodology and theoretical bases to explore the applications of intelligent physical robots with various functions across different health care contexts. We propose some future agendas for research on intelligent physical robots in the health care context from conceptual, thematic, methodological, and technological perspectives.

#### Conceptual Agenda

In future research, it is vital to have a broad and comprehensive conceptual understanding of what an intelligent physical robot is. As the field is still in a nascent state, different terms have been used to describe similar concepts. With the integration and development of robots and AI technologies, robots have begun to be endowed with varying degrees of intelligence, and they have been called intelligent robots, social robots, and chatbots [119]. The different functions of robots and the diversity of their use contexts also make it difficult to have a unified classification of robots. Therefore, more research is needed to clearly identify the distinctions and boundaries between different robots and to build a clear and consistent conceptual framework to define them.

#### Thematic Agenda

Our findings have shown that individual-related, organization-related, and robot-related factors can affect individuals’ acceptance of intelligent physical robots in the health care context. Previous research has focused more on exploring the individual-related factors in intelligent physical robot use in health care, and few existing studies have explored the organization-related and robot-related antecedents of intelligent physical robot use in the health care context. In addition, most studies have focused on the motivations for intelligent physical robot use, and few have attempted to examine the barriers to intelligent physical robot use. Furthermore, previous studies have examined the outcomes of non–health-related outcomes and health promotion, but there is a lack of studies explaining how humans should interact or cooperate with robots in various health care contexts to achieve these outcomes identified in this study. On the basis of these findings, we proposed the following thematic agenda.

First, user acceptance of intelligent physical robots in health care is important for their successful application in the health care context [109,112,113]. Previous research has focused on the demographic and social characteristics of individual users. Future research needs to consider the barriers to robot use, such as users’ privacy, ethics, and risk concerns, and their negative emotions, which can provide an understanding of robot use by examining both motivators and barriers.

Second, future research should consider intelligent physical robot implementation in health care organizations from the viewpoint of digital transformation at the organizational level. Previous research has highlighted the effectiveness and efficiency of robots in health care services [31,58,62] but has ignored the digital transformation brought about by intelligent physical robot implementation in health care organizations. It is necessary to evaluate intelligent physical robot implementation in health care organizations from a wide perspective, such as from the view of digital transformation, to evaluate its value in health care.

Third, future research should further investigate the effects of different anthropomorphic features of intelligent physical robots on users in different health care contexts. Previous studies have examined various aspects of robot design, but there is a lack of understanding of the relationship between anthropomorphism and user perceptions, attitudes, and behaviors in the health care context. More research is needed to provide knowledge on the effects of intelligent physical robot design in the health care context and on how robot use can meet different needs of different user groups.

Fourth, in the included studies, few have considered how humans should cooperate with robots to realize their benefits. Thus, future research should explore human-robot interaction in intelligent physical robot use to provide knowledge on how humans could benefit from interacting with these robots. With the trend toward robots and humans working together, we should consider how to collaborate with intelligent physical robots to find the right way for humans and robots to interact in harmony.

#### Methodological Agenda

To gain a deep understanding of the use of intelligent physical robots in health care, a variety of research methods should be applied in future research. For example, big data analysis, focus group interviews, and action research should be considered as complementary research methods when researching the use of intelligent physical robots in the health care context. In addition, in the dominant quantitative research methods, such as experimentation and surveys, the sample group should be expanded to different user groups and should not be limited to older adults and female caregivers. Furthermore, comparative and longitudinal studies could be considered in future research to improve the diversity and effectiveness of the research design.

#### Technological Agenda

Future research on intelligent physical robot design in the health care context should consider the interactive capabilities of robots in creating a natural and realistic service experience. One of the important characteristics of intelligent robots is their capacity for interaction. However, many participants in the included studies raised concerns regarding the limited interactive capabilities of robots [5,74]. This not only requires intelligent robots to improve their perception of humans but also needs humans to be provided with a rich interactive experience. We suggest exploring how to improve the technological design of robots. In addition, future research needs to consider technical security and data privacy in robot design.

### Limitations

It is important to acknowledge the limitations of this study. First, we searched the articles from 5 databases using some keywords, which may have limited the selection of articles and excluded some relevant articles that did not contain these applied keywords in their abstracts and titles or were published in other databases. Future work could expand the scope of the database and search terms to include more relevant articles in literature reviews in the field. Second, 8 studies with low-quality research methodologies according to the MMAT were included in the review. Thus, there might be a potential risk of bias in our findings based on the literature review. Future research could consider including only articles with high quality in research methodologies in literature reviews in the field. Third, some studies had a small sample size or included research participants from a specific geographical area, which may have potentially affected the data representativeness in our findings. Future research could consider including articles with a broader geographical and population diversity. Fourth, this study focuses on identifying the antecedents and consequences of intelligent physical robot use in the health care context, and other aspects related to intelligent physical robots, such as human-robot interaction and collaboration, are worthy of further research.

### Conclusions

This study has made several contributions to the literature. First, it provides an overview of the antecedents and consequences of intelligent physical robot use in the health care context by synthesizing and analyzing the current literature on intelligent physical robots in the health care context. Specifically, this study contributes to the literature by identifying individual-related factors (eg, social and psychological factors), organization-related factors (environment and resource view), and robot-related factors (eg, robot design and function) as antecedents of intelligent physical robot use and non–health-related consequences (emotional outcomes, attitude and evaluation outcomes, and behavioral outcomes regarding technology use), and consequences for (physical, mental, and social) health promotion. Second, this study proposed an integrative framework to synthesize the antecedents and consequences of intelligent physical robot use in the health care context, which provides scholars with an integrated overview of the antecedents and consequences of intelligent physical robot use in the health care context. Finally, the literature review helps identify some gaps in the research on intelligent physical robots in the health care context and provides scholars with some future research directions from the perspectives of concepts, methodologies, research themes, and robot technologies. Thus, this study also provides guidance to scholars for identifying future research topics regarding intelligent physical robot use in the health care context, which could potentially address existing research gaps and improve research in the field.

## Appendix

Multimedia Appendix 1 PRISMA (Preferred Reporting Items for Systematic Reviews and Meta-Analyses) checklist.

Multimedia Appendix 2 Search strategies in each database.

Multimedia Appendix 3 Details of the included studies.

Multimedia Appendix 4 Mixed Methods Appraisal Tool quality assessment report.

Multimedia Appendix 5 Contexts in the included studies.

Multimedia Appendix 6 Robotic devices in the included studies.

Multimedia Appendix 7 Target users in the included studies.

Multimedia Appendix 8 Antecedents of robot use in health care in the included studies.

Multimedia Appendix 9 Consequences of robot use in health care in the included studies.

## Abbreviations

AI: artificial intelligence
MMAT: Mixed Methods Appraisal Tool
PRISMA: Preferred Reporting Items for Systematic Reviews and Meta-Analyses
