# Supplementary material for: Intelligent Physical Robots in Health Care: Systematic Literature Review
Source: J Med Internet Res. 2023 Jan 18;25:e39786. doi: 10.2196/39786 (PMC9892988; doi:10.2196/39786)
Supplement: Multimedia Appendix 2 [file jmir_v25i1e39786_app2.docx]

**Appendix 2.** **Search strategies in each database**

Comprehensive search strategy: (healthcare OR “health care” OR nursing) AND (robot* OR bot)

Search time: May 2021

Search strategies in five databases:

--------------------------------------------------------------------------------

**PubMed search strategy**

| Search | Query | Results |
| --- | --- | --- |
| #1 | ((healthcare[Title/Abstract]) OR ("health care"[Title/Abstract])) OR (nursing[Title/Abstract]) | 801261 |
| #2 | ("robot*"[Title/Abstract] OR "bot"[Title/Abstract]) | 51577 |
| #3 | #1 AND #2 | 1400 |
| #4 | #1 AND #2 (Filters: published in English) | 1342 |
| Note: [Title/Abstract] is the combined search field that searches abstracts and titles of articles in PubMed. | | |

--------------------------------------------------------------------------------

**Scopus search strategy**

| Search | Query | Results |
| --- | --- | --- |
| #1 | ( ( TITLE-ABS ( healthcare ) OR TITLE-ABS ( "health care" ) OR TITLE-ABS ( nursing ) ) | 2,638,438 |
| #2 | ( TITLE-ABS ( robot* ) OR TITLE-ABS ( bot ) ) | 574,974 |
| #3 | #1 AND #2 | 4511 |
| #4 | #1 AND #2 (Filters: published in English) | 4361 |
| Note: TITLE-ABS is the combined search field that searches abstracts and titles of articles in Scopus. | | |

--------------------------------------------------------------------------------

**PsycInfo search strategy**

| Search | Query | Results |
| --- | --- | --- |
| #1 | abstract(healthcare) OR abstract("health care") OR abstract(nursing) | 78,498 |
| #2 | abstract(robot*) OR abstract(bot) | 1685 |
| #3 | #1 AND #2 | 275 |
| #4 | #1 AND #2 (Filters: published in English) | 275 |
| Note: Abstract is the search field that searches abstracts of articles in PsycInfo. | | |

--------------------------------------------------------------------------------

**EMBASE search strategy**

| Search | Query | Results |
| --- | --- | --- |
| #1 | (healthcare:ab,ti OR 'health care':ab,ti OR nursing:ab,ti) | 995,819 |
| #2 | (robot*:ab,ti OR bot:ab,ti) | 75,850 |
| #3 | #1 AND #2 | 1973 |
| #4 | #1 AND #2 (Filters: published in English) | 1551 |
| Note: :ab,ti is the combined search field that searches abstracts and titles of articles in EMBASE. | | |

--------------------------------------------------------------------------------

**CINAHL search strategy**

| Search | Query | Results |
| --- | --- | --- |
| #1 | AB healthcare OR AB "health care" OR AB nursing | 486,349 |
| #2 | AB robot* OR AB bot | 9,398 |
| #3 | #1 AND #2 | 570 |
| #4 | #1 AND #2 (Filters: published in English) | 530 |
| Note: AB is the search field that searches abstracts of articles in CINAHL. | | |
