# Supplementary material for: Intelligent Physical Robots in Health Care: Systematic Literature Review
Source: J Med Internet Res. 2023 Jan 18;25:e39786. doi: 10.2196/39786 (PMC9892988; doi:10.2196/39786)
Supplement: Multimedia Appendix 3 [file jmir_v25i1e39786_app3.docx]

**Appendix 3. Details of the included studies**

| **Reference** | **Study method** | **Publication type** | **Theoretical base** | **Robotic platform** | **Context** | **Main finding** |
| --- | --- | --- | --- | --- | --- | --- |
| [3] | Case study (n=5) | Conference | / | iRobi | Rural town | The results showed a decrease in primary care visits and phone calls to practitioners while the robot was present and increases in quality of life were observed. |
| [4] | Survey (n=499) | Journal | / | / | Community centers | Robots have the potential to help with the caregiving and domestic needs of the growing aging population and of older adults with multiple chronic conditions. |
| [5] | Experiment (n=59) | Conference | / | CuDDler | Older care | High ratings in “likeability” and “perceived safety”. Weakness (average score): anthropomorphism, animacy, and perceived intelligence. |
| [6] | Interview (n=28) | Journal | / | Silbot | Large retirement facility | Both older adults and experts thought the six activities would be useful for people with mild cognitive impairment and mild dementia and possibly provide companionship, stimulation, and reassurance, and reduce caregiver burden. |
| [10] | Experiment (n=40) | Journal | / | EveR-4 | University | Both smiling and use of the first name had significant positive effects on participants’ perceptions of robot personality. |
| [11] | Interview (n=18) | Journal | / | / | Older care organizations | Three use cases were mentioned as the most promising: the robot as a ubiquitous aid; the robot as a helper in the room; and the robot as a guide. |
| [12] | Observational study (n=27) | Journal | / | Nao | Nursing home, day care center | Older people could successfully exercise with the assistance of the robot. They did not prefer the robots as an exercise tutor over the human coach. |
| [13] | Experiment (n=2) | Journal | / | / | Health center | Participants were satisfied with the line-following capabilities and the liquid crystal display interaction of robots. |
| [14] | Ethnographic methods (n=19) | Journal | / | Paro | Large urban hospital | Patient participants perceived the robot Paro as helpful in supporting the psychosocial needs for inclusion, identity, attachment, and comfort. |
| [15] | Mixed method (survey n=108 and interview n=108) | Journal | The unified theory of acceptance and use of technology | / | Home healthcare agencies | The usage intention of home healthcare robots is a function of social influence, performance expectancy, trust, privacy concerns, ethical concerns, and facilitating conditions. |
| [16] | Experiment (n=51) | Conference | / | Pepper, Erica and Sophia | / | How human-like the robot is affects older people’s preferences in favor of android robots. Older people expressed a clear preference for female android rather than humanoid robots. |
| [17] | Experiment (n=23) | Journal | / | Paro | Long-term care facility | Use of Paro resulted in increased observed positive affective and behavioral indicators, with concomitant decreases observed in negative affective and behavioral indicators. Paro is best presented to residents who are relatively calm and approachable. |
| [18] | Survey (n=416) | Journal | / | / | Long-term care facility | Most health personnel had positive attitudes towards the use of social robots in long-term care facilities, as they viewed social robots as beneficial and practical in psychosocial care for older adults. |
| [20] | Experiment (n=29) | Conference | / | iRobiQ; Cafero | Retirement village | The robots were found to be acceptable and feasible, and many participants described them as useful and as friends although not all comments were positive. |
| [30] | Experiment (n=57) | Conference | / | Charles | Home | The results showed few differences between the two age groups. Males have a more positive attitude towards robots in healthcare than females. |
| [31] | Survey (n=331) | Journal | / | / | Hospital | Robots’ ability to focus nurses’ efforts on professional tasks may help improve nurses’ health and overall job satisfaction, and by extension reduce their turnover. |
| [32] | Mixed method (qualitative and quantitative strand (n=19) | Journal | IT affordance | / | Care facility | Five niches were identified: service robots as means of reducing the workloads of healthcare professional, strategic assets for tackling spending problems, specialist tools for supporting highly skilled hospital professionals, gadgets with unknown practical value, and a means of boosting the quality of services and information. |
| [33] | Experiment (n=99) | Conference | / | charlie | Retirement village | Older people in private places used the healthcare service for checking their health conditions, and older people in public places like to use the entertainment services. |
| [34] | Experiment (n=57) | Journal | / | Peoplebot | University | People who think that robots are human-like experience heightened wariness in interactions with robots. |
| [35] | Survey (n=302) | Journal | / | / | Hospitals | Trial center nurses and those with more than 10-year experience have positive perceptions on robotic care. The top‐three desired primary roles of robotic care are measuring/monitoring, mobility/activity, and safety care. |
| [36] | Experiment (n=133) | Conference | / | charlie | Rural medical clinic | Average medical consultation lengths were reduced when a robot was used to take patients' vital signs in a rural medical clinic. Use of the robot was found to be cost-effective based on its projected use for 20% of consultations. |
| [37] | Experiment (n=3) | Conference | / | Kabochan, Pepper | Psychiatric hospital and older institution | Interactions with Pepper robot were often not spontaneous and mediation from human staff was integral to successful interactions between older adults and Pepper. |
| [42] | Experiment (n=24) | Journal | Technology acceptance model | Adaptive Robotic Nurse Assistant | Simulated hospital environment | Participants had positive opinions in general and did not completely agree that use of the Adaptive Robotic Nurse Assistant improves their job performance or saves them a substantial amount of time. |
| [43] | Survey (n=1004) | Journal | User acceptance models | / | Japan, Ireland, and Finland | In Japan, people's perspectives on home-care robots were mostly related to safety. In Ireland, most of the respondents wanted to help other people and society by participating in the research and development of home-care robots. Finland had the highest percentage of respondents who said that healthcare professionals should be allowed to use secondary information collected by a home-care robot. |
| [44] | Survey (n=466) | Journal | Technology acceptance model; Theory of Mind | / | Hospital | The staff’s intentions and facilitation of support played a key role in adopting and using robots. |
| [45] | Survey (n=25) | Journal | Technology acceptance model; Theory of mind | Charlie | Retirement village | Residents who held significantly more positive attitudes towards robots, and who perceived robot minds to have less agency, were more likely to use the robot. Attitudes towards robots improved over time in robot-users. |
| [46] | Experiment (n=3) | Journal | Applied behavior analysis | Nao | University-based applied behaviour analysis clinic | Almost all the children improve in their ability to answer wh- questions. |
| [47] | Survey (n=200) | Journal | Theory of planned behavior | / | Home care facility | Examination of the attitudes towards robots of home care personnel combines the perspectives of societal attitudes, attitudes related to psychological reactions, and the practical care and promotion of the independent living of older people. |
| [48] | Experiment (n=164) | Journal | Theory of Planned Behavior | / | Laboratory | The genders and personalities of social robots interact with corresponding role stereotypes to affect user acceptance of social robots. |
| [49] | Experiment (n=10) | Journal | Activity engagement | Brain 2.0 | Laboratory | The robot Brian 2.0 with social interaction capabilities will increase the likelihood of a person engaging in a specific cognitively stimulating activity. |
| [50] | Experiment (n=34) | Journal | Emotional well-being | Matilda | Residential care facilities | Matilda positively engages older people in group and one-to-one activities, helping them become resilient and cope better through personalization of care. |
| [51] | Experiment (n=132) | Journal | Emotional appraisal theory | Alice | Care facility | Manipulated coping potential indirectly affected perceptions of a healthcare robot via the appraisal of coping potential. Positive emotion-focused coping affected perceptions of a healthcare robot positively. |
| [52] | Interview (n=7) | Conference | Adoption theories; The capability approach | Nao | Home | The transfer effects of assistive robots from the older persons’ perspective are to convert the functional (physical, visual, and memory) and non-functional (psychological) support provided by robots into everyday life capabilities (housework, mobility, reading, remembering, and interactions, etc.). |
| [53] | Observational study | Journal | The Transactive Relationship Theory of Nursing | Pepper | Kagawa Prefecture | It was considered important that healthcare providers play roles as intermediaries in order smoothly to carry out conversations with Pepper with the application program of care prevention gymnastics exercises. |
| [54] | Experiment (n=60) | Journal | / | Paro | Nursing homes | This study found a long-term effect on depression and agitation as a result of using Paro in activity groups for older people with dementia in nursing homes. |
| [55] | Experiment (n=100) | Journal | / | / | Nursing home | The dogs and the robot seal stimulated the residents to more interaction, compared with the toy cat, but the robot seal failed to hold attention at the same level over time. |
| [56] | Experiment (n=30 dyads) | Journal | / | Paro | Dementia day care centers | Paro shows promise in enhancing affective and social outcomes for certain individuals with dementia. People with greater cognitive capacity seemed to respond more positively to Paro. |
| [57] | Experiment (n=5) | Journal | / | Paro | Residential mental healthcare institution | Robot-based automating academic tasks does not have clear beneficial effects on alertness and mood in adults with moderate to severe identity, but positive interactions with the robot seal could be of therapeutic value in themselves. |
| [58] | Experiment (n=42) | Journal | / | Pepper | Clinical outpatient | Social robots may effectively and acceptably assist healthcare professionals by interviewing older adults. |
| [59] | Experiment (n=87) | Journal | / | Paro | Local community | Children who interacted with the robot showed greater increases in positive mood, but did not differ from control participants in terms of negative mood, anxiety, or arousal. |
| [60] | Mixed method (focus groups n=27 and survey n=260) | Conference | / | / | Home | The acceptance of a domestic robot seems to be rather strong. Participants prefer the non-personal care of a robot in situations of bodily exposure compared with the caring process that involves the affective side. |
| [61] | Experiment (n=30) | Journal | / | Peoplebot | University | The more human-like a healthcare robot’s face display is, the more people attribute mind and positive personality characteristics to it. Eeriness was related to negative impressions of the robot’s personality. |
| [62] | Experiment (n=31) | Journal | / | Pepper | Community | Patient-reported outcome measures data collection in older persons may be carried out effectively and efficiently by a social robot. |
| [63] | Experiment (n=171) | Journal | / | Healthbot | Older care facility | Participants prefer an empathetic voice (emotions in the voice and variations in prosody) from a robotic companion compared to a robotic voice (non-emotional, with an inappropriate tone and a lack of friendliness in the voice) in a healthcare application; |
| [64] | Experiment (n=91) | Journal | / | EveR-4 | Flu vaccination scenario | Robot use of humor resulted in significantly greater perceptions of the robot’s likeability and safety. |
| [65] | Experiment (n=16) | Conference | / |  | Group campus | Children reacts very positively towards the robots and would like to interact with the robot in a manner that the current setup is not able to deliver. |
| [66] | Experiment (n=2) | Conference | / | Human support robot | Users' home | Human support robot provides potential benefit to severely disabled people who cannot move their arms, but there still is some criticism of manual mode, pick-up, and fetch task. |
| [67] | Mixed method (focus group n=18 and survey n=39) | Journal | / | Paro | Nursing homes and a geriatric hospital | Positive opinions about Paro and high expectations of professionals regarding Paro: facilitation of nursing care, creation of a distraction, and reduction of patients’ aggressive behaviors. |
| [68] | Experiment (n=65) | Conference | / | iRobiQ | University | Young people rated the robot's usefulness and ease of use quite highly and thought that reminders to take medication and measurement of vital signs would be most useful for older people, and also somewhat useful for children, teenagers and young adults. |
| [69] | Mixed method (interview n=14 and survey n=14) | Conference | / | Personal Robot 2 | Hospital | Participants would be likely to accept assistance from a personal robot like the Personal Robot 2 for instrumental activities of daily living, followed by activities of daily living, medical tasks and administration/communication tasks. |
| [70] | Mixed method (survey n=62 and interview n=62) | Conference | / | Hopis and In-touch | Retirement center | Residents are more positive about robots than staff, and participants prefer a silver robot of 1.25 m height, with wheels and a screen on the body. |
| [71] | Experiment (n=43) | Conference | / | iRobiQ | University | The brain game is enjoyable for young people, although young people thought the quality of the brain game should be improved. They liked the robot dancing to music videos, and thought that it is a useful function. |
| [72] | Mixed method (survey n=3800 and interview n=969) | Journal | / | / | Healthcare institutions | Previous experiences with robots were consistently correlated with robot acceptance. Robot assistance was welcomed for certain tasks (heavy lifting and logistics). |
| [73] | Experiment (n=53) | Conference | / | Healthbot | Retirement village | Pre-interaction emotions and attitudes towards robots, as well as experience with the robot, are important areas to monitor and address in influencing acceptance of healthcare robots in retirement village residents and staff. |
| [74] | Mixed method (interview n=9 and survey n=9) | Journal | / | Paro | Geriatric psychiatry departments | Healthcare providers perceived Paro as an effective intervention to improve the well-being of people with dementia (provide distraction, interrupt problematic behaviors, stimulate communication). |
| [75] | Survey (n=286) | Journal | / | / | Care homes | Japanese care personnel assessed the usefulness of robots more positively than did their Finnish counterparts. There are also certain fears (replacement of people, dehumanization of treatment, and increased loneliness in the older group), in particular among the Finnish care personnel. |
| [76] | Survey (n=444) | Journal | / | home care robot | Japanese prefecture | While perceived benefit was the common predictor affecting the willingness of home-care staff to use a robot for the care of all user types, concerns regarding the use of personal information were more prominent for older people. |
| [77] | Interview (n=10) | Conference | / | CareBot, RIBA Robot, Paro | Older care facilities | Seven themes were identified: 1) Knowledge about robots; 2) Attitude to companion robot; 3) Attitude to lifting robot; 4) Attitude to therapy robot; 5) Cannot replace human contact; 6) Explicit ethical issues; 7) Whether robots should be given responsibility. |
| [78] | Experiment (n=14) | Journal | / | Pepper | Retirement village | A robot showing emotions in a didactic setting may not, by default, negatively influence participants' acceptance and perception of the robot. An older person may not become distressed if the robot breaks or is taken away from them, as attachment to the robot in this didactic setting was not strong. |
| [79] | Interview (n=123) | Conference | / | Companion robots | University | Users were not convinced that the robot on its own could effectively encourage independence-promoting behavior. |
| [80] | Survey (n=102) | Conference | / | Medicine delivery robot | Older care facility | The contradiction of inherent trust and simultaneous wariness about control gives rise to the following phenomenon: older people with needs want control over their care to ensure it is personalized, but many desperately take any help they can get. |
| [81] | Survey (n=357) | Journal | / | Assistant, monitoring, and companion robots | University | The acceptance of robots in care was more strongly associated with the participants’ moral considerations than with utility. |
| [82] | Survey (n=58) | Journal | / | Charles | Care facility | Participants’ perceptions about robots were influenced by their prior exposure to robots in literature or entertainment media. Participants felt more comfortable with a medical student and saw the robot as less accurate. |
| [83] | Mixed method (interview n=35 and survey n=62) | Journal | / | Zora | Care organizations | Zora robot had a positive influence on clients as it created added value for the care professionals in having fun at work. |
| [84] | Experiment (n=11) | Conference | Servicescape framework | / | / | Equipment and design & deco were the general and essential conditions when designing a robot. Space and ambience were subjects that should have greater importance from the viewpoint of actual application in a healthcare environment. |
| [85] | Experiment (n=90) | Conference | / | Pepper, Romeo | / | Androids were clearly more preferred by the older adults from Campania, Italy, than humanoid robots, regardless of gender traits. |
| [86] | Experiment (n=11) | Journal | / | Paro | Older care facility | Residents with dementia expressed positive attitudes towards the use of Paro and acknowledged the therapeutic benefits of Paro on mood improvement and relaxation for pain relief. |
| [87] | Experiment (n=35) | Conference | / | Nao | Residential and clinical facility | The impressions given by the caregivers were consistently positive, not only about the effect of the robot on their workload, but also about the positive effects of the robots on a user's engagement with the activity. |
| [88] | Case study (n=7) | Journal | / | Lio | Care facility | Older people and health care staff are very curious and open towards the robot. Female voices were perceived to be friendlier, but male voices were easier for older people to understand. |
| [89] | Interview (n=10) | Conference | / | / | Large outpatient healthcare provider | Older adults are willing and able to participate in design projects for social assistive robot. |
| [90] | Experiment (n=18) | Journal | / | Paro | Older care facility | Paro had a moderate to large positive influence on participants’ quality of life compared to the control group. |
| [91] | Experiment (n=23) | Journal | / | Robovie-R3 | Older care facility | Walking with the robot evoked the participants’ perception of novelty or stimulated an existing interest in assistive robotics, both leading to positive feelings. |
| [92] | Experiment (n=10) | Conference | / | Healthbot | Older care facility | The older adults could complete the tasks successfully, felt confident while using the system, and found it easy and simple. |
| [93] | Case study (n=9) | Conference | / | Max | Home | The older people appreciated the robot's health-related instrumental functions and they even built emotional bonds with it. |
| [94] | Experiment (n=40) | Journal | / | Paro | Residential care facility | Paro has benefits on reducing loneliness for older people in nursing home care and has an effect on the social environment comparable to that of a live animal. |
| [95] | Survey (n=19) | Conference | / | Paro | Older care institutions | Most of the caregivers were familiar with robot-assisted therapy and generally quite willing to apply it if they did not already. |
| [96] | Experiment (n=41) | Journal | / | Paro | Australian nursing homes | At week one, residents in the Paro group had a greater increase in the night sleep period. At week six, residents in the Paro group showed a greater increase in daytime wakefulness and a greater reduction in daytime sleep. |
| [97] | Interview (n=43) | Conference | / | Tangy | Long-term care facilities | Participants provided positive feedback about these activities, and suggested a variety of additional functionalities. |
| [98] | Experiment (n=6) | Conference | / | Brian 2.0 | Laboratory | The social interaction capabilities of Brian 2.0 are effective in engaging individuals in a cognitively stimulating game. |
| [99] | Experiment (n=43) | Journal | / | Paro | Long-term care facilities | Participants in the Paro group had a significantly lowered level of observed pain and used fewer medications. |
| [100] | Experiment (n=12) | Conference | / | Paro | Laboratory | A therapy session using animal robot Paro has huge potential to reduce symptoms of depression such as stress and insomnia. |
| [101] | Experiment (n=14) | Journal | / | Pepper | Clinic | The robot was received with more enthusiasm by the older adults, thus improving their level of engagement. |
| [102] | Experiment (n=142) | Conference | / | / | / | The situation in which a robot interacts with a human affected the emotions of the older participants differently: in a service situation, fewer negative emotions were expressed than in a care situation. |
| [103] | Experiment (n=100) | Journal | / | Paro | Nursing home | The dog and the interactive robot seal triggered the most interaction, but the robot failed to hold attention at the same level over time. |
| [104] | Survey (n=2365) | Journal | / | / | University | All the students viewed companion robots as more beneficial than monitoring and assistive robots, but they rated use intentions low, indicating a poor motivation to actually use a robot in the future (fear of job loss). |
| [105] | Experiment (n=70) | Conference | / | Nao | Home | Children perceived Nao as a someone who could be with them; not only to help them or provide support about diabetes, but mainly to share daily life experiences and difficulties. |
| [106] | Experiment (n=78) | Journal | / | Cota and Palro | Residential care homes | The overall findings support the use of com‐robots within the context of a care team for older people with moderate dementia, and those who are more than 80 years old. |
| [107] | Interview (n=3) | Journal | / | Paro | Older care facility | The findings support the use of Paro as a therapeutic tool, revealing improvement in emotional state, reduction of challenging behaviors, and improvement in the social interactions of residents. |
| [108] | Interview (n=23) | Journal | / | / | Pediatric unit | Care impact-advantages: (a) reducing healthcare providers' workload; (b) adequately meeting the needs of pediatric patients and their families. Shortcomings: (a) deficiency of individualized care; (b) reduced employment opportunities for skilled nursing staff. |
| [109] | Experiment (n=48) | Journal | Technology acceptance model | Brain 2.1 | Long-term care facility | Participants accepted the robot and had positive attitudes towards its assistive and social capabilities. |
| [110] | Interview (n=20) | Journal | / | / | / | Robotic pets may be an effective solution for alleviating loneliness in older adults, especially among those who live alone, have fewer social connections, and live fewer active lifestyles. |
| [111] | Experiment (n=26) | Journal | / | Guide Paro | Secure dementia residential facility | Paro was found to be more acceptable to family members, staff, and residents. Healthcare robots in dementia settings have to be simple and easy to use as well as stimulating and entertaining. |
| [112] | Mixed method (focus group n=21 and survey n=89) | Journal | / | / | Retirement village | Concerns included the loss of jobs and personal care, while perceived benefits included allowing staff to spend quality time with residents, and helping residents with self-care. |
| [113] | Survey (n=178) | Journal | / | / | University | Generally positive attitudes of future medical staff (view robot as an assistant rather than as a companion for older people). |
