# Supplementary material for: Intelligent Physical Robots in Health Care: Systematic Literature Review
Source: J Med Internet Res. 2023 Jan 18;25:e39786. doi: 10.2196/39786 (PMC9892988; doi:10.2196/39786)
Supplement: Multimedia Appendix 4 [file jmir_v25i1e39786_app4.docx]

**Appendix 4. Mixed Methods Appraisal Tool (MMAT) quality assessment report**

**Table 1. Mixed Methods Appraisal Tool (MMAT), version 2018.**

| **Category of study designs** | **Methodological quality criteria** |
| --- | --- |
|  |  |
| Screening questions  (for all types) | S1. Are there clear research questions? |
|  | S2. Do the collected data allow to address the research questions? |
| 1. Qualitative method | 1.1. Is the qualitative approach appropriate to answer the research question? |
|  | 1.2. Are the qualitative data collection methods adequate to address the research question? |
|  | 1.3. Are the findings adequately derived from the data? |
|  | 1.4. Is the interpretation of results sufficiently substantiated by data? |
|  | 1.5. Is there coherence between qualitative data sources, collection, analysis and interpretation? |
| 2. Quantitative randomized controlled trials | 2.1. Is randomization appropriately performed? |
|  | 2.2. Are the groups comparable at baseline? |
|  | 2.3. Are there complete outcome data? |
|  | 2.4. Are outcome assessors blinded to the intervention provided? |
|  | 2.5 Did the participants adhere to the assigned intervention? |
| 3. Quantitative non-randomized trials | 3.1. Are the participants representative of the target population? |
|  | 3.2. Are measurements appropriate regarding both the outcome and intervention (or exposure)? |
|  | 3.3. Are there complete outcome data? |
|  | 3.4. Are the confounders accounted for in the design and analysis? |
|  | 3.5. During the study period, is the intervention administered (or exposure occurred) as intended? |
| 4. Quantitative descriptive trials | 4.1. Is the sampling strategy relevant to address the research question? |
|  | 4.2. Is the sample representative of the target population? |
|  | 4.3. Are the measurements appropriate? |
|  | 4.4. Is the risk of nonresponse bias low? |
|  | 4.5. Is the statistical analysis appropriate to answer the research question? |
| 5. Mixed methods | 5.1. Is there an adequate rationale for using the mixed methods design to address the research question? |
|  | 5.2. Are the different components of the study effectively integrated to answer the research question? |
|  | 5.3. Are the outputs of the integration of qualitative and quantitative components adequately interpreted? |
|  | 5.4. Are divergences and inconsistencies between quantitative and qualitative results adequately addressed? |
|  | 5.5. Do the different components of the study adhere to the quality criteria of each tradition of the methods involved? |
| Note: To appraise 5.5, use criteria for the qualitative component (1.1-1.5), and the appropriate criteria for the quantitative component (2.1-2.5, or 3.1-3.5, or 4.1-4.5). | |

**Table 2. Quality of the included studies (n=94)**

| **Reference** | **Qualitative** | | | | | **Quantitative RCTs** | | | | | **Quantitative nonrandomized** | | | | | **Quantitative descriptive** | | | | | **Mixed methods** | | | | | **Total score** |
| --- | --- | --- | --- | --- | --- | --- | --- | --- | --- | --- | --- | --- | --- | --- | --- | --- | --- | --- | --- | --- | --- | --- | --- | --- | --- | --- |
|  | 1 | 2 | 3 | 4 | 5 | 1 | 2 | 3 | 4 | 5 | 1 | 2 | 3 | 4 | 5 | 1 | 2 | 3 | 4 | 5 | 1 | 2 | 3 | 4 | 5 |  |
| [3] | - | - | - | - | - | - | - | - | - | - | Y | Y | Y | ? | Y | - | - | - | - | - | - | - | - | - | - | 4 |
| [4] | - | - | - | - | - | - | - | - | - | - | - | - | - | - | - | Y | Y | Y | Y | Y | - | - | - | - | - | 5 |
| [5] | Y | Y | ? | N | Y | - | - | - | - | - | - | - | - | - | - | - | - | - | - | - | - | - | - | - | - | 3 |
| [6] | Y | Y | Y | Y | Y | - | - | - | - | - | - | - | - | - | - | - | - | - | - | - | - | - | - | - | - | 5 |
| [10] | - | - | - | - | - | Y | Y | Y | ? | Y | - | - | - | - | - | - | - | - | - | - | - | - | - | - | - | 4 |
| [11] | Y | Y | ? | N | Y | - | - | - | - | - | - | - | - | - | - | - | - | - | - | - | - | - | - | - | - | 3 |
| [12] | - | - | - | - | - | - | - | - | - | - | - | - | - | - | - | Y | N | Y | ? | Y | - | - | - | - | - | 3 |
| [13] | - | - | - | - | - | - | - | - | - | - | - | - | - | - | - | ? | N | Y | Y | Y | - | - | - | - | - | 3 |
| [14] | Y | Y | Y | Y | Y | - | - | - | - | - | - | - | - | - | - | - | - | - | - | - | - | - | - | - | - | 5 |
| [15] | Y | Y | Y | Y | Y | - | - | - | - | - | - | - | - | - | - | Y | Y | Y | ? | Y | N | Y | Y | ? | Y | 3 |
| [16] | - | - | - | - | - | - | - | - | - | - | - | - | - | - | - | Y | Y | Y | ? | Y | - | - | - | - | - | 4 |
| [17] | - | - | - | - | - | - | - | - | - | - | - | - | - | - | - | Y | Y | Y | Y | Y | - | - | - | - | - | 5 |
| [18] | - | - | - | - | - | - | - | - | - | - | - | - | - | - | - | Y | Y | Y | ? | Y | - | - | - | - | - | 4 |
| [20] | - | - | - | - | - | N | Y | Y | Y | Y | - | - | - | - | - | - | - | - | - | - | - | - | - | - | - | 4 |
| [30] | - | - | - | - | - | - | - | - | - | - | Y | Y | Y | Y | Y | - | - | - | - | - | - | - | - | - | - | 5 |
| [31] | - | - | - | - | - | - | - | - | - | - | - | - | - | - | - | Y | Y | Y | Y | Y | - | - | - | - | - | 5 |
| [32] | - | - | - | - | - | - | - | - | - | - | Y | Y | Y | ? | Y | - | - | - | - | - | - | - | - | - | - | 4 |
| [33] | - | - | - | - | - | - | - | - | - | - | - | - | - | - | - | Y | Y | Y | ? | Y | - | - | - | - | - | 4 |
| [34] | - | - | - | - | - | - | - | - | - | - | - | - | - | - | - | ? | Y | Y | Y | Y | - | - | - | - | - | 4 |
| [35] | - | - | - | - | - | - | - | - | - | - | - | - | - | - | - | Y | Y | Y | N | Y | - | - | - | - | - | 4 |
| [36] | - | - | - | - | - | - | - | - | - | - | Y | Y | Y | ? | Y | - | - | - | - | - | - | - | - | - | - | 4 |
| [37] | - | - | - | - | - | - | - | - | - | - | Y | Y | Y | N | Y | - | - | - | - | - | - | - | - | - | - | 4 |
| [42] | - | - | - | - | - | N | ? | Y | ? | Y | - | - | - | - | - | - | - | - | - | - | - | - | - | - | - | 2 |
| [43] | Y | N | Y | Y | Y | - | - | - | - | - | - | - | - | - | - | - | - | - | - | - | - | - | - | - | - | 4 |
| [44] | - | - | - | - | - | - | - | - | - | - | Y | Y | Y | N | Y | - | - | - | - | - | - | - | - | - | - | 4 |
| [45] | - | - | - | - | - | - | - | - | - | - | - | - | - | - | - | Y | Y | Y | Y | Y | - | - | - | - | - | 5 |
| [46] | - | - | - | - | - | - | - | - | - | - | Y | Y | Y | N | N | - | - | - | - | - | - | - | - | - | - | 3 |
| [47] | - | - | - | - | - | N | Y | Y | Y | Y | - | - | - | - | - | - | - | - | - | - | - | - | - | - | - | 4 |
| [48] | - | - | - | - | - | Y | Y | N | Y | N | - | - | - | - | - | - | - | - | - | - | - | - | - | - | - | 3 |
| [49] | - | - | - | - | - | - | - | - | - | - | N | Y | ? | N | ? | - | - | - | - | - | - | - | - | - | - | 1 |
| [50] | Y | ? | Y | Y | ? | - | - | - | - | - | - | - | - | - | - | ? | Y | Y | Y | Y | N | N | Y | ? | N | 1 |
| [51] | - | - | - | - | - | N | ? | Y | ? | Y | - | - | - | - | - | - | - | - | - | - | - | - | - | - | - | 2 |
| [52] | - | - | - | - | - | - | - | - | - | - | - | - | - | - | - | ? | N | Y | ? | Y | - | - | - | - | - | 2 |
| [53] | - | - | - | - | - | N | Y | Y | ? | Y | - | - | - | - | - | - | - | - | - | - | - | - | - | - | - | 3 |
| [54] | - | - | - | - | - | Y | Y | Y | N | ? | - | - | - | - | - | - | - | - | - | - | - | - | - | - | - | 3 |
| [55] | - | - | - | - | - | - | - | - | - | - | Y | Y | Y | ? | Y | - | - | - | - | - | - | - | - | - | - | 4 |
| [56] | - | - | - | - | - | ? | Y | Y | ? | Y | - | - | - | - | - | - | - | - | - | - | - | - | - | - | - | 3 |
| [57] | - | - | - | - | - | - | - | - | - | - | Y | Y | Y | N | ? | - | - | - | - | - | - | - | - | - | - | 3 |
| [58] | - | - | - | - | - | N | Y | Y | Y | Y | - | - | - | - | - | - | - | - | - | - | - | - | - | - | - | 4 |
| [59] | - | - | - | - | - | Y | Y | Y | ? | Y | - | - | - | - | - | - | - | - | - | - | - | - | - | - | - | 4 |
| [60] | Y | ? | Y | Y | Y | - | - | - | - | - | - | - | - | - | - | ? | Y | Y | Y | Y | Y | Y | Y | ? | Y | 4 |
| [61] | - | - | - | - | - | - | - | - | - | - | - | - | - | - | - | ? | Y | Y | Y | Y | - | - | - | - | - | 4 |
| [62] | - | - | - | - | - | - | - | - | - | - | N | Y | Y | Y | ? | - | - | - | - | - | - | - | - | - | - | 3 |
| [63] | - | - | - | - | - | - | - | - | - | - | - | - | - | - | - | Y | Y | Y | ? | Y | - | - | - | - | - | 4 |
| [64] | - | - | - | - | - | - | - | - | - | - | - | - | - | - | - | N | Y | Y | ? | Y | - | - | - | - | - | 3 |
| [65] | - | - | - | - | - | - | - | - | - | - | Y | Y | Y | ? | Y | - | - | - | - | - | - | - | - | - | - | 4 |
| [66] | Y | N | Y | Y | Y | - | - | - | - | - | - | - | - | - | - | - | - | - | - | - | - | - | - | - | - | 4 |
| [67] | Y | N | Y | Y | Y | - | - | - | - | - | - | - | - | - | - | Y | Y | Y | ? | Y | Y | Y | Y | ? | Y | 4 |
| [68] | - | - | - | - | - | - | - | - | - | - | - | - | - | - | - | Y | Y | Y | ? | Y | - | - | - | - | - | 4 |
| [69] | - | - | - | - | - | - | - | - | - | - | Y | Y | Y | ? | ? | - | - | - | - | - | - | - | - | - | - | 3 |
| [70] | Y | Y | Y | Y | Y | - | - | - | - | - | - | - | - | - | - | Y | Y | Y | Y | Y | N | Y | Y | ? | Y | 3 |
| [71] | - | - | - | - | - | - | - | - | - | - | - | - | - | - | - | ? | Y | Y | ? | Y | - | - | - | - | - | 3 |
| [72] | - | - | - | - | - | - | - | - | - | - | - | - | - | - | - | Y | Y | Y | Y | Y | - | - | - | - | - | 5 |
| [73] | - | - | - | - | - | - | - | - | - | - | - | - | - | - | - | Y | Y | Y | ? | Y | - | - | - | - | - | 4 |
| [74] | Y | N | Y | Y | Y | - | - | - | - | - | - | - | - | - | - | Y | Y | Y | Y | Y | Y | Y | Y | ? | Y | 4 |
| [75] | - | - | - | - | - | - | - | - | - | - | Y | Y | Y | N | Y | - | - | - | - | - | - | - | - | - | - | 4 |
| [76] | - | - | - | - | - | - | - | - | - | - | - | - | - | - | - | Y | Y | Y | N | Y | - | - | - | - | - | 4 |
| [77] | Y | N | Y | Y | Y | - | - | - | - | - | - | - | - | - | - | - | - | - | - | - | - | - | - | - | - | 4 |
| [78] | - | - | - | - | - | - | - | - | - | - | - | - | - | - | - | ? | Y | Y | Y | Y | - | - | - | - | - | 4 |
| [79] | Y | Y | Y | Y | Y | - | - | - | - | - | - | - | - | - | - | - | - | - | - | - | - | - | - | - | - | 5 |
| [80] | Y | N | Y | Y | Y | - | - | - | - | - | - | - | - | - | - | Y | N | Y | N | Y | Y | Y | Y | ? | N | 3 |
| [81] | - | - | - | - | - | - | - | - | - | - | - | - | - | - | - | Y | Y | Y | Y | Y | - | - | - | - | - | 5 |
| [82] | - | - | - | - | - | - | - | - | - | - | - | - | - | - | - | Y | Y | Y | Y | Y | - | - | - | - | - | 5 |
| [83] | Y | Y | Y | Y | Y | - | - | - | - | - | - | - | - | - | - | Y | Y | Y | ? | Y | N | Y | Y | ? | Y | 3 |
| [84] | Y | N | Y | Y | Y | - | - | - | - | - | - | - | - | - | - | - | - | - | - | - | - | - | - | - | - | 4 |
| [85] | - | - | - | - | - | - | - | - | - | - | Y | Y | Y | ? | Y | - | - | - | - | - | - | - | - | - | - | 4 |
| [86] | Y | Y | Y | N | ? | - | - | - | - | - | - | - | - | - | - | - | - | - | - | - | - | - | - | - | - | 3 |
| [87] | - | - | - | - | - | - | - | - | - | - | Y | Y | Y | ? | Y | - | - | - | - | - | - | - | - | - | - | 4 |
| [88] | - | - | - | - | - | - | - | - | - | - | - | - | - | - | - | Y | N | Y | ? | Y | - | - | - | - | - | 3 |
| [89] | Y | N | Y | Y | Y | - | - | - | - | - | - | - | - | - | - | - | - | - | - | - | - | - | - | - | - | 4 |
| [90] | Y | Y | Y | Y | Y | - | - | - | - | - | - | - | - | - | - | Y | Y | Y | ? | Y | Y | Y | Y | Y | Y | 5 |
| [91] | - | - | - | - | - | ? | Y | N | Y | N | - | - | - | - | - | - | - | - | - | - | - | - | - | - | - | 2 |
| [92] | - | - | - | - | - | - | - | - | - | - | - | - | - | - | - | Y | Y | Y | ? | Y | - | - | - | - | - | 4 |
| [93] | Y | ? | Y | N | Y | - | - | - | - | - | - | - | - | - | - | ? | Y | Y | ? | Y | N | Y | Y | ? | N | 2 |
| [94] | - | - | - | - | - | N | Y | Y | Y | Y | - | - | - | - | - | - | - | - | - | - | - | - | - | - | - | 4 |
| [95] | - | - | - | - | - | - | - | - | - | - | - | - | - | - | - | ？ | Y | Y | Y | Y | - | - | - | - | - | 4 |
| [96] | - | - | - | - | - | - | - | - | - | - | - | - | - | - | - | Y | Y | Y | ? | Y | - | - | - | - | - | 4 |
| [97] | Y | Y | Y | Y | Y | - | - | - | - | - | - | - | - | - | - | - | - | - | - | - | - | - | - | - | - | 5 |
| [98] | - | - | - | - | - | - | - | - | - | - | Y | Y | N | ? | Y | - | - | - | - | - | - | - | - | - | - | 3 |
| [99] | Y | N | Y | Y | Y | - | - | - | - | - | - | - | - | - | - | - | - | - | - | - | - | - | - | - | - | 4 |
| [100] | - | - | - | - | - | - | - | - | - | - | Y | Y | N | N | ? | - | - | - | - | - | - | - | - | - | - | 2 |
| [101] | Y | Y | Y | Y | Y | - | - | - | - | - | - | - | - | - | - | - | - | - | - | - | - | - | - | - | - | 5 |
| [102] | - | - | - | - | - | - | - | - | - | - | - | - | - | - | - | ? | Y | Y | Y | Y | - | - | - | - | - | 4 |
| [103] | Y | Y | Y | Y | Y | - | - | - | - | - | - | - | - | - | - | Y | Y | Y | ? | Y | N | Y | Y | ? | Y | 3 |
| [104] | - | - | - | - | - | - | - | - | - | - | Y | Y | Y | ? | Y | - | - | - | - | - | - | - | - | - | - | 4 |
| [105] | Y | Y | Y | Y | Y | - | - | - | - | - | - | - | - | - | - | - | - | - | - | - | - | - | - | - | - | 5 |
| [106] | Y | N | Y | Y | Y | - | - | - | - | - | - | - | - | - | - | - | - | - | - | - | - | - | - | - | - | 4 |
| [107] | Y | N | Y | Y | Y | - | - | - | - | - | - | - | - | - | - | - | - | - | - | - | - | - | - | - | - | 4 |
| [108] | Y | Y | Y | Y | Y | - | - | - | - | - | - | - | - | - | - | - | - | - | - | - | - | - | - | - | - | 5 |
| [109] | - | - | - | - | - | N | Y | Y | ? | Y | - | - | - | - | - | - | - | - | - | - | - | - | - | - | - | 3 |
| [110] | Y | Y | Y | Y | Y | - | - | - | - | - | - | - | - | - | - | - | - | - | - | - | - | - | - | - | - | 5 |
| [111] | - | - | - | - | - | - | - | - | - | - | - | - | - | - | - | Y | Y | Y | N | Y | - | - | - | - | - | 4 |
| [112] | Y | Y | Y | Y | Y | - | - | - | - | - | - | - | - | - | - | Y | Y | Y | N | Y | N | Y | Y | ? | Y | 3 |
| [113] | - | - | - | - | - | - | - | - | - | - | - | - | - | - | - | ? | Y | Y | Y | Y | - | - | - | - | - | 4 |
