# Supplementary material for: Intelligent Physical Robots in Health Care: Systematic Literature Review
Source: J Med Internet Res. 2023 Jan 18;25:e39786. doi: 10.2196/39786 (PMC9892988; doi:10.2196/39786)
Supplement: Multimedia Appendix 5 [file jmir_v25i1e39786_app5.docx]

# **Appendix 5-A. Contexts in the included studies**

| **Research context (amounts of articles)** | **Source** |
| --- | --- |
| Older care facility (n=42) | [5-6, 11-13, 15, 17-18, 32, 37, 43, 47, 50-51, 54-55, 63, 67, 70, 72, 75, 77, 80, 82, 83, 86-88, 90-93, 95, 97, 99, 102-103, 106-107, 109, 111] |
| University/campus (n=11) | [10, 34, 61, 64-65, 68, 71, 79, 81, 104, 113] |
| Retirement village (n=9) | [3, 20, 33, 53, 45, 73, 76, 78, 112] |
| Hospital (n=7) | [31, 35, 42, 44, 67, 69, 84] |
| Laboratory (n=7) | [12, 16, 48-49, 85, 98, 100] |
| Clinic/outpatient (n=6) | [36, 46, 58, 89, 101, 108] |
| Home (n=6) | [30, 52, 60, 66, 94, 105] |
| Mental care institution (n=5) | [14, 37, 56-57, 74] |
| Community (n=4) | [4, 59, 62, 110] |
| Note: Three studies relate to two contexts; thus, the sum is larger than 94.  **Appendix 5-B Contexts in the included studies** | |
