# Supplementary material for: Intelligent Physical Robots in Health Care: Systematic Literature Review
Source: J Med Internet Res. 2023 Jan 18;25:e39786. doi: 10.2196/39786 (PMC9892988; doi:10.2196/39786)
Supplement: Multimedia Appendix 6 [file jmir_v25i1e39786_app6.docx]

# **Appendix 6-A. Robotic devices in the included studies.**

| **Robot type (amounts of articles)** | **Platforms** | **Physical characteristics** | **Primary purpose** | **Source** |
| --- | --- | --- | --- | --- |
| Mechanoid (n=17) | Adaptive Robotic Nurse Assistant, Guide, Charlie, Charles, Healthbot, Peoplebot, Carero, HSR, In-touch, Lio, Max and Personal Robot 2 | In the form of a service kiosk, usually with a mobile body, touch screen, camera, and handlebar | Nursing assistant | [34, 36, 42, 45, 61, 63, 83, 92, 111] |
|  |  |  | Support activities of daily living | [30, 33, 45, 66, 69-70, 73, 88, 93] |
| Animalistic (n=20) | Paro, Hopis, RIBA and CuDDler | Animal-like appearance, such as bear or seal | Support cognitive therapy | [14, 17, 54, 56-57, 59, 67, 74, 77, 86, 90, 94-96, 99, 100, 103, 107] |
|  |  |  | Support activities of daily living | [70, 77] |
|  |  |  | Provide social companion | [5] |
| Humanoid (n=26) | Pepper, Nao, Brain, Zora, Cota, Palro, Kabochan, Tangy, Sophia, Matilda, Robovie-R3, Romeo, Silbot and iRobiQ | Human-like appearance with head, neck, and limbs | Support cognitive therapy | [12, 37, 46, 49, 52, 87, 97-98, 101, 105-106] |
|  |  |  | Provide social companion | [16, 50] |
|  |  |  | Support activities of daily living | [3, 6, 20, 68, 71, 78, 83, 85, 91, 109] |
|  |  |  | Patient interview | [58, 62] |
|  |  |  | Support physical therapy | [53] |
| Android (n=4) | Alice, Erica and EveR-4 | Rather realistic human-like appearance, usually composed of a flesh-like material | Provide social companion | [16, 51] |
|  |  |  | Patient reception and communication | [10, 64] |

# **Appendix 6-B. Robot types in the included studies.**
