# Supplementary material for: Intelligent Physical Robots in Health Care: Systematic Literature Review
Source: J Med Internet Res. 2023 Jan 18;25:e39786. doi: 10.2196/39786 (PMC9892988; doi:10.2196/39786)
Supplement: Multimedia Appendix 7 [file jmir_v25i1e39786_app7.docx]

# **Appendix 7. Target users in the included studies.**

| **User type (amounts of articles)** | **Target users**  **(amounts of articles)** | **Source** |
| --- | --- | --- |
| **End-customer (n=75)** | Older adults (n=38) | [3-4, 6, 12, 17, 30, 33, 36-37, 43, 45, 50, 52-53, 55, 58, 62-63, 70, 73, 78-79, 85-86, 89-94, 96, 102-103, 106-107, 109, 110, 112] |
|  | Adults with cognitive impairment (n=10) | [14, 54, 56-57, 67, 98-99, 100-101, 111] |
|  | University students and staff (n=8) | [10, 48, 51, 61, 64, 68, 71, 104] |
|  | Healthy adults (n=5) | [5, 12, 16, 30, 49, 80] |
|  | Patients (n=4) | [10, 13, 34, 36] |
|  | Children (n=4) | [46, 59, 65, 105] |
|  | Patients' relatives (n=4) | [75, 97, 105, 111] |
|  | People with limited arm or leg mobility (n=2) | [66, 100] |
| **Healthcare professionals (n=65)** | Medical doctors (n=5) | [6, 15, 18, 32, 44] |
|  | Nursing professionals (n=20) | [6, 15, 18, 31-32, 35, 42, 44, 47, 67, 69-70, 72, 76, 81, 84, 87, 97, 108, 113] |
|  | Personal care and home care workers (n=16) | [43, 70, 73-74, 76-77, 79, 81, 82-83, 87, 89, 95, 97, 111, 112] |
|  | Healthcare technicians and assistants (n=5) | [15, 18, 67, 69, 82] |
|  | Health management personnel (n=11) | [6, 11, 15, 18, 32, 70, 72, 76, 83, 97, 112] |
|  | Other healthcare professionals (n=8) | [11, 15, 18, 44, 67, 70, 72, 76] |
| Note. Several studies relate to two or more target users; thus, the sum is larger than 94. | | |
