# Supplementary material for: Intelligent Physical Robots in Health Care: Systematic Literature Review
Source: J Med Internet Res. 2023 Jan 18;25:e39786. doi: 10.2196/39786 (PMC9892988; doi:10.2196/39786)
Supplement: Multimedia Appendix 8 [file jmir_v25i1e39786_app8.docx]

# **Appendix 8. Antecedents of robot use in health care in the included studies**

| **Category**  **(amount of articles)** | **Sub-category** | **Factors** | **Source** |
| --- | --- | --- | --- |
| **Individual-related factors**  **(n=37)** | End-customer | Demographic factors (age, gender and nationality) | [30, 43, 68, 71, 85] |
|  |  | Psychological factors (perception, motivation and attitude) | [3, 5-6, 14, 17, 20, 33, 36, 42, 45, 51-52, 56, 66, 68, 71, 73, 80, 92, 103, 112] |
|  |  | Experience-related factors (experience with robots, exposed duration) | [20, 45, 82] |
|  | Healthcare professional | Demographic factors | [44, 72, 75-76] |
|  |  | Psychological factors | [4, 6, 11, 42, 44, 53, 73-76, 81, 112] |
|  |  | Experience-related factors (experience with robots, working experience) | [35, 44, 72, 76, 83] |
| **Organization-related factors**  **(n=12)** | Environment | Physical environment (building structure, atmosphere, and decoration) | [14, 32-33, 84, 102] |
|  | Resource | Top management support | [44, 72, 83, 112] |
|  |  | Personnel readiness | [14, 35, 44, 67, 83, 104] |
|  |  | IT infrastructure | [32, 83-84] |
| **Robot-related factors**  **(n=20)** | Design | Appearance (weight, gender, ethnicity) | [16, 34, 48, 61, 85-86, 112] |
|  |  | Voice | [63, 67, 87, 88] |
|  |  | Personality | [6, 10, 48, 61, 64, 87, 88] |
|  | Function | Cognitive capacity | [74, 88] |
|  |  | Navigation capability | [88] |
|  |  | Interactive capability | [10, 87-88] |
|  |  | Automatic responses/updates | [33, 74, 83] |
|  |  | Software failures | [56, 84, 89] |
|  | | | |
