# Supplementary material for: Intelligent Physical Robots in Health Care: Systematic Literature Review
Source: J Med Internet Res. 2023 Jan 18;25:e39786. doi: 10.2196/39786 (PMC9892988; doi:10.2196/39786)
Supplement: Multimedia Appendix 9 [file jmir_v25i1e39786_app9.docx]

# **Appendix 9. Consequences of robot use in health care in the included studies**

| **Category**  **(amounts of articles)** | **Sub-category** | **Factors** | **Source** |
| --- | --- | --- | --- |
| **Non-health related consequences**  **(n=72)** | Emotional outcomes | Pleasure | [90] |
|  |  | Satisfaction | [13, 31] |
|  |  | Wariness | [34, 61] |
|  |  | Likeability | [5] |
|  |  | Enjoyment | [71, 91] |
|  | attitude and evaluation outcomes | Negative opinions / concerns about robots' appearance, capability, and impacts | [11, 35, 60, 66, 69, 74-75, 82-83, 86-87, 89, 104, 108, 112-113] |
|  |  | Perception of the roles of robots in healthcare | [10-11, 32, 43, 52, 65, 105, 108, 113] |
|  |  | Preference and suggestions for robots' appearance, function, applied tasks and situation | [6, 12-13, 16, 30, 33, 35, 60, 66, 68-70, 75, 87, 95, 97, 112] |
|  |  | Positive attitude /views on robotic care | [3-5, 10, 20, 30, 33, 35, 48, 58, 60, 63, 69, 71-73, 75, 82, 87, 89, 104, 108, 111, 112-113] |
|  | Behavioral outcomes | Willing to use | [15, 43, 76, 88, 93, 95, 104, 110-111] |
|  |  | Willing to interact | [16, 85, 89, 100] |
|  |  | Use frequency and duration | [36, 55, 58, 67, 71, 92, 96, 109, 111] |
|  |  | Task performance | [12-13, 46, 62, 66, 82, 91, 101, 109] |
|  |  | Response and engagement with robots | [6, 10, 14, 17, 33, 37, 49, 50, 54-56, 58, 65, 74, 83, 98, 101, 103, 105, 110] |
| **Consequences for health promotion**  **(n=39)** | Physical health | Improving medication adherence regarding physical health | [3, 20, 99] |
|  |  | Prompting rehabilitation exercise | [12, 53, 92] |
|  |  | Supporting independent living on activities of daily living | [6, 68, 82, 93, 105, 109] |
|  | Mental health | Improving medication adherence regarding mental health | [3, 20, 54, 56, 99-100] |
|  |  | Improving mental mood | [10, 17, 57, 59, 107] |
|  |  | Improving cognitive capabilities | [46, 49, 57, 98, 101] |
|  |  | Improving behavioral and psychological symptoms of dementia | [17, 20, 54, 56, 59, 74, 86, 90, 96, 99-100, 102] |
|  | Social health | Prompting engagement in social activities | [6, 50, 92, 109] |
|  |  | Facilitating social connections with others | [55-56, 74, 94, 103, 106-107] |
|  |  | Companionship | [6, 66, 85, 94, 104, 110] |
